# S100 proteins: a new frontier in fibromyalgia research

**DOI:** 10.1186/s13041-024-01102-9

**Published:** 2024-05-26

**Authors:** María Teresa Vega-Ramírez, Enrique Becerril-Villanueva, José Luis Maldonado-García, Lenin Pavón, Gilberto Pérez-Sánchez

**Affiliations:** 1 Laboratorio de Psicoinmunología, Instituto Nacional de Psiquiatría Ramón de la Fuente Muñiz, Colonia San Lorenzo Huipulco, Tlalpan, Ciudad de México, 14370 México; 2 Departamento de Inmunología, Escuela Nacional de Ciencias Biológicas, Instituto Politécnico Nacional, Miguel Hidalgo, Ciudad de México, México; 3 Departamento de Bioquímica, Facultad de Medicina, Universidad Nacional Autónoma de México, Coyoacán, Ciudad de México, México

## Abstract

Fibromyalgia (FM) is a chronic condition that causes widespread pain, fatigue, and other symptoms that significantly affect quality of life. The underlying mechanisms of fibromyalgia involve both the immune system and the central nervous system. It has been proposed that changes in multiple ascending and descending pathways in the central nervous system may contribute to increased pain sensitivity in individuals with this condition. Recent research has identified S100 proteins as a new area of interest in fibromyalgia studies. These proteins are a group of small molecular weight proteins involved in inflammation and various functions inside and outside of cells, and they may play a critical role in the development and progression of FM. Although S100B has been the most studied in FM patients, other studies have reported that S100A7, S100A8, S100A9, and S100A12 may also be useful as potential biomarkers or for a deeper understanding of FM pathophysiology. The potential role of S100 proteins in the pathophysiology of fibromyalgia could be mediated by RAGE and TLR4, which signal through JNK, ERK, and p38 to activate AP-1 and NF-κB and induce the release of proinflammatory cytokines, thereby producing the inflammation, fatigue, and chronic pain characteristic of fibromyalgia. To gain new perspectives on targeted therapeutic approaches, it is crucial to understand how S100 proteins could impact the pathophysiology of fibromyalgia. This review examines the potential role of S100 proteins in fibromyalgia and their impact on improving our comprehension of the condition, as well as facilitating further research on this interesting topic.

## Introduction

One of the main features of fibromyalgia is the heightened sensitivity to pain, known as hyperalgesia. This hyperalgesia could be explained in part by central sensitization, which refers to an abnormal amplification of pain signals within the central nervous system. S100 proteins, particularly S100A8 and S100A9, are significantly upregulated in inflammatory conditions and have been shown to mediate inflammatory responses and pain perception. Thus, S100 protein-mediated inflammation could be a key element in the pathophysiology of FM. This review presents the S100 proteins that have been reported as dysregulated in patients with fibromyalgia and discusses their potential contribution to the pathophysiology of FM.

### Dysregulated S100 proteins in patients with fibromyalgia: clinical reports

In patients with fibromyalgia, S100A8 (calgranulin A) and S100A12 (calgranulin C) were found to be upregulated in saliva samples [1], while the expression of S100A8 and S100A9 was found to be increased in peripheral B cells of these patients [2]. S100B is the most studied S100 protein in patients with this chronic condition, and it has been found to be significantly elevated in serum of women with several chronic pain conditions, including fibromyalgia [3, 4]. In contrast, the serum levels of S100A7, were found to be lower in FM patients than in controls [5]. The disruption of these S100 proteins in individuals diagnosed with fibromyalgia suggests that these proteins play a pivotal role in the pathogenesis of this disease.

### Potential mechanisms of S100 proteins in fibromyalgia pathophysiology

**Fig. 1:**
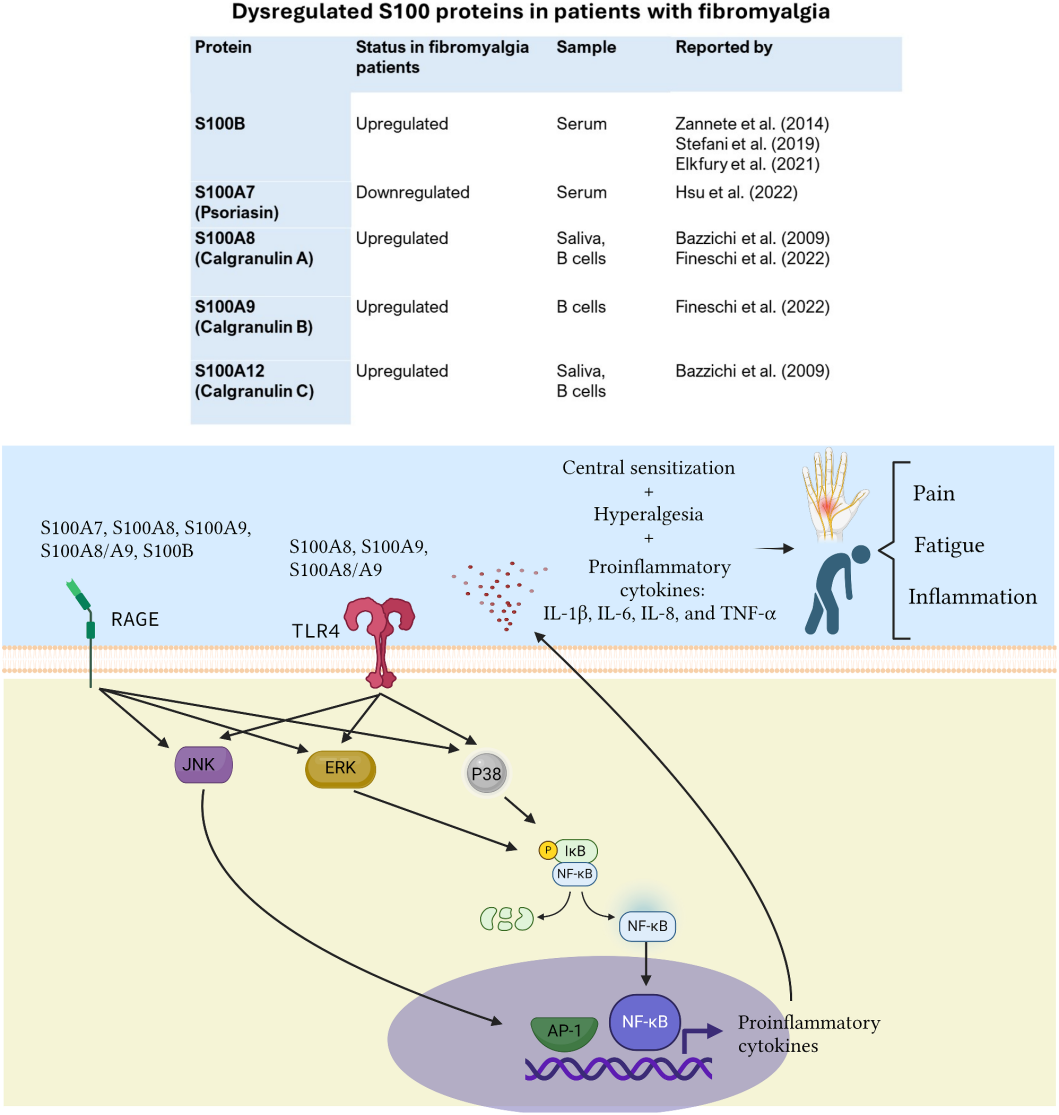
The potential role of S100 proteins in the pathophysiology of fibromyalgia. S100A8, S100A9, S100A8/A9, S10012, and S100B bind to RAGE, while S100A8, S100A8, and S100A8/A9 dimer bind to toll-like receptor (TLR4). Both RAGE and TLR4 signal through JNK, ERK, and p38 to activate the transcription factors AP-1 (Activating Protein-1) and NF-κB (nuclear factor kappa B), which in turn induce the release of proinflammatory cytokines. These proinflammatory cytokines, coupled with the presence of hyperalgesia and central sensitization, may contribute to inflammation, fatigue and chronic pain

When released extracellularly, S100 proteins can act as damage-associated molecular patterns (DAMPs) and contribute significantly to inflammatory pain. It is well established that S100A7, S100A8, S100A9, S100A8/A9, S10012, and S100B bind to RAGE, while S100A8, S100A8, and S100A8/A9 dimer bind to toll-like receptor (TLR4). Both RAGE and TLR4 are expressed in cells such as macrophages and signal through JNK, ERK, and p38 to activate the transcription factors AP-1 (Activating Protein-1) and NF-κB (nuclear factor kappa B), which in turn induce the release of proinflammatory cytokines [6–8]. It has been demonstrated that increased proinflammatory cytokines, such as IL-1β, IL-6, IL-8, and TNF-α, are associated with the development of neuropathic pain and fatigue. IL-6 has been shown to alter the resting membrane potential threshold and cause hyperexcitability in neurons, which is secondary to phosphorylation of the sodium channel Nav1.7. This results in an increase in the transmission of pain signals. Furthermore, IL-6 has been implicated in the development of pain related to changes in neuronal plasticity [9, 10]. In a previous study, it was reported that serum concentrations of IL-6 and IL-8 were increased in FM patients. These cytokines exhibited a positive correlation with clinical scores on the Fibromyalgia Impact Questionnaire (FIQ), suggesting that IL-6 and IL-8 may have additive or synergistic effects in perpetuating the chronic pain experienced by FM patients [11]. Taken together, it is possible that the activity of S100 proteins is associated with the generation of pain through the release of proinflammatory cytokines (Fig. 1).

S100 proteins have also been considered as potential biomarkers for both CNS damage and brain tumors [11]. In addition, S100 proteins have been associated with changes in neuronal electrical properties, suggesting a possible role in neuronal sensitization [12]. Interestingly, S100A4 has also been implicated in the modulation of neuropathic pain in rodent models [13]. It is important to note that chronic pain is linked to neural plasticity, which refers to changes in pain transmission and perception in the CNS [14]. Therefore, the increased levels of S100 proteins, particularly S100B (together with BDNF), may be associated with changes in neuronal plasticity, which translates to chronic pain in patients. This is particularly relevant when discussing hyperalgesia and central sensitization. The increased perception of pain caused by hyperalgesia could be partially explained by central sensitization, which amplifies pain signals within the CNS. Central sensitization causes temporal, spatial and threshold alterations in pain sensitivity and is a clear example of the contribution of the CNS to the generation of pain hypersensitivity. Central sensitization enhances the function of neurons and circuits in nociceptive pathway, resulting in an increased response of the somatosensory nervous system in response to inflammation and neural injury [15]. When considering the contribution of S100 proteins to the inflammatory state, the pathophysiology of FM becomes more complex, since we must consider that these patients are potentially more susceptible to inflammatory processes. The pathophysiology of fibromyalgia involves a non-virtuous cycle between hyperalgesia, central sensitization, and S100 protein-mediated inflammation. This cycle significantly contributes to one of the most characteristic symptoms of fibromyalgia, which is chronic widespread pain. Figure 1 illustrates the potential role of S100 proteins in the pathophysiology of fibromyalgia.

## Conclusion

Exploration of S100 proteins as a target of fibromyalgia therapy opens up the possibility of developing a totally novel strategy. In essence, by modulating the function of S100 proteins we could potentially reverse the core symptoms of fibromyalgia, which include features of inflammation, pain, and neuroinflammation. Prospective therapeutic strategies can be designing drugs that interfere with S100 proteins’ interactions or treatments that keep the expression of S100 proteins in check. Concerning the role of S100 proteins on the pathophysiology of FM, it needs a deeper exploration from a molecular point of view; however, this initial information is important to guide future research.

## Data Availability

Not applicable for this type of manuscript.

## Abbreviations

FM: Fibromyalgia
CNS: Central Nervous System
PPT: Pressure-Pain Threshold
BDNF: Brain-derived neurotrophic factor
DAMPs: Damage-associated molecular patterns
RAGE: Receptor for advanced glycation end products
TLR: Toll-like receptor
